# Protocol to decode the role of transcriptionally active microbes in SARS-CoV-2-positive patients using an RNA-seq-based approach

**DOI:** 10.1016/j.xpro.2024.103071

**Published:** 2024-05-19

**Authors:** Aanchal Yadav, Priti Devi, Pallawi Kumari, Ranjeet Maurya, Uzma Shamim, Rajesh Pandey

**Affiliations:** 1 Division of Immunology and Infectious Disease Biology, INtegrative GENomics of HOst-PathogEn (INGEN-HOPE) Laboratory, CSIR-Institute of Genomics and Integrative Biology (CSIR-IGIB), Mall Road, Delhi 110007, India; 2 Academy of Scientific and Innovative Research (AcSIR), Ghaziabad 201002, India; 3 Indraprastha Institute of Information Technology (IIIT), New Delhi 110020, India

**Keywords:** Health Sciences, Genomics, Microbiology

## Abstract

The elucidation of the role of microorganisms in human infections has been hindered by difficulties using conventional culture-based techniques. Here, we present a protocol for the investigation of transcriptionally active microbes (TAMs) using an RNA sequencing (RNA-seq)-based approach. We describe the steps for RNA isolation, viral genome sequencing, RNA-seq library preparation, and metatranscriptomic and transcriptomic analysis. This protocol permits a comprehensive evaluation of TAMs’ contributions to the differential severity of infectious diseases, with a particular focus on diseases such as COVID-19.

For complete details on the use and execution of this protocol, please refer to Devi et al.^1^

## Highlights

• A protocol to characterize transcriptionally active microbes (TAMs) using RNA-seq
• Steps for RNA-seq library preparation, sequencing, and metatranscriptomic analysis
• Detailed procedure for taxonomic and functional classification of microbes
• Correlation between bacterial species and the expressed host genes

## Abstract (editor highlights)

Publisher’s note: Undertaking any experimental protocol requires adherence to local institutional guidelines for laboratory safety and ethics.

## Graphical abstract

**Figure:**
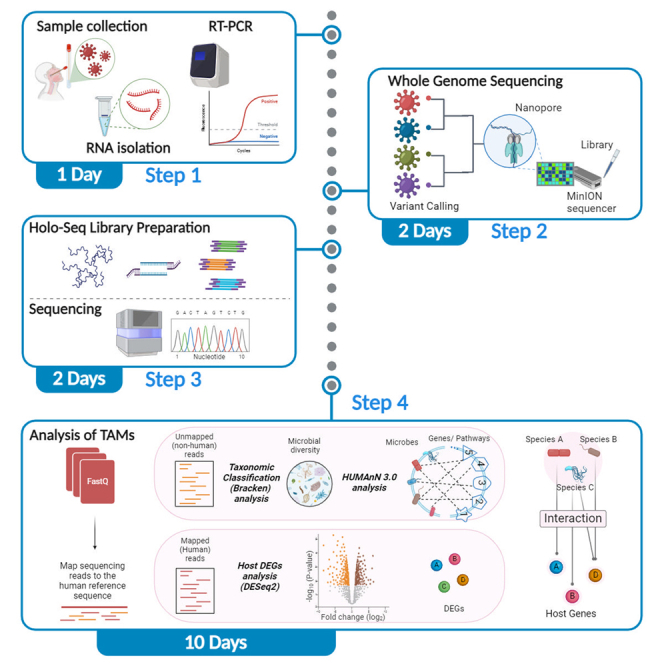

## Before you begin

Comprehending the significance of TAMs in influencing the course or outcome of a disease represents a crucial aspect of clinical research that has not received in-depth examination thus far. This protocol outlines the precise steps required for conducting a functional metagenomics study among the various SARS-CoV-2 variants of concern (VOCs) from the nasopharyngeal swab isolated from the COVID-19 positive patients. These same procedures are applicable for investigating the metagenomic profile of any other sample/disease type. In case of blood samples, it is imperative to first eliminate globin mRNA along with the ribosomal RNA before proceeding with the preparation of the RNA-seq library.

### Nasopharyngeal swab collection (performed by a trained healthcare provider, only)

The trained paramedical staff at the MAX Hospital, Delhi collect the nasopharyngeal swabs of patients on the day of reporting to the hospital. Put the tip of the swab into a vial containing 3 mL of Viral Transport Media (VTM) (HiViral Transport Kit, HiMedia, Cat. No: MS2760A-50NO), by breaking the applicator’s stick and sealing the tube tightly. Vortex the tube for 2 min to allow the dissolution of the sample into the VTM solution followed by centrifugation and allow it to settle for some time before processing.

### Preparation of running the codes

#### Guppy installation

**Timing: 10 min**

1. Login to Nanopore community website (https://community.nanoporetech.com) using nanopore login credential, login id and password (https://community.nanoporetech.com/).
2. Direct to “Software downloads” page from right selection options (https://community.nanoporetech.com/downloads).
3. Download Guppy (latest v6.5.7) for Linux 64-bit GPU download option.
4. Install Guppy to GPU enabled server.

#### R and RStudio installation

**Timing: 20–30 min**

5. The latest version of R can be found at https://cran.r-project.org/. This is the official website for the R programming language, where you can download the latest version and related packages.
6. The installation of RStudio is not mandatory to follow the protocol. However, it makes viewing and interacting with files, packages, objects in the environment, tables, and graphs easy. RStudio can be found at https://posit.co/download/rstudio-desktop/ and can greatly enhance your R programming experience by providing a user-friendly interface for working with R scripts and data.

#### Installation of tools/softwares/packages before analysis

**Timing: 1 h**

conda install -c bioconda::fastqc

conda install -c bioconda::trimmomatic

conda install -c bioconda::hisat2

conda install -c bioconda::samtools

conda install -c bioconda::kraken2

conda install -c bioconda::bracken

conda install -c bioconda::humann

***Note:*** Set up the “conda” package manager using the official link https://conda.io/projects/conda/en/latest/user-guide/getting-started.html for creating and activating the conda environment.

## Key resources table

**Table:** 

REAGENT or RESOURCE	SOURCE	IDENTIFIER
**Critical commercial assays**		

Viral transport medium (VTM)	HiViral Transport Kit, HiMedia	Cat. No. MS2760A-50NO
Viral RNA extraction	QIAmp viral mini kit, QIAGEN	Cat. No. 52906
TRUPCR SARS-CoV-2 kit	3B BlackBio Biotech India Ltd.	Cat. No. 3B304
LunaScript RT SuperMix Kit	New England Biolabs (NEB)	Cat. No. E3010
Q5 Hot Start High-Fidelity 2X master mix	NEB	Cat. No. M0494
NEBNext Ultra II End Repair/dA – tailing module	NEB	Cat. No. E7546
Blunt/TA ligase master mix	NEB	Cat. No. M0367
NEBNext Quick ligation module	NEB	Cat. No. E6056
Ligation Sequencing Kit	Oxford Nanopore Technologies	SQK-LSK109
Native Barcoding Expansion 96	Oxford Nanopore Technologies	EXP-NBD196
Sequencing flow cells	Oxford Nanopore Technologies	FLO-MIN106 (R9.4.1)
Flow Cell Wash Kit	Oxford Nanopore Technologies	EXP-WSH004
TruSeq Stranded Total RNA Library Prep Gold	Illumina	Cat. No. 20020599
IDT for Illumina – TruSeq RNA UD indexes (96 indexes, 96 samples)	Illumina	Cat. No. 20022371
AMPure XP	Beckman Coulter	Cat. No. A63881
Agencourt RNAClean XP Kit	Beckman Coulter	Cat. No. A63987
Qubit dsDNA HS Assay Kit	Symbio (Thermo Fisher Scientific)	Cat. No. Q32854
Bioanalyzer DNA high sensitivity kit	Agilent	Cat. No. 5067-4626

**Deposited data**		

RNA-seq data	This study, NCBI Sequence Read Archive (SRA) database	NCBI SRA: PRJNA676016, PRJNA678831 (Pre-VOC), PRJN868733, PRJNA952815 (VOCs)

**Software and algorithms**		

R (v4.3.3)	CRAN	https://cran.r-project.org/bin/windows/base/
RStudio	Posit	https://posit.co/download/rstudio-desktop/
bcl2fastq (v2.19.0.316)	GitHub	https://github.com/brwnj/bcl2fastq
Guppy (v2.1.0)	Oxford Nanopore Technologies	https://community.nanoporetech.com/
Minimap2 (v2.17)	Li, Heng^2^	https://github.com/lh3/minimap2
Nanopolish (v0.14.1)	Loman et al.^3^	https://github.com/jts/nanopolish
FastQC (v0.12.0)	GitHub	https://github.com/s-andrews/FastQC
Trimmomatic (v0.39)	Bolger et al.^4^	http://www.usadellab.org/cms/?page=trimmomatic
HISAT2	Kim et al.^5^	https://github.com/DaehwanKimLab/hisat2
Samtools (1.17)	Danecek et al.^6^	https://github.com/samtools/
Kraken2 (v2.0.8)	Wood et al.^7^	https://github.com/DerrickWood/kraken2/wiki
Bracken2 (v2.7.0)	Lu et al.^8^	https://github.com/jenniferlu717/Bracken
HUMAnN3	Beghini et al.^9^	https://github.com/biobakery/humann?tab=readme-ov-file
metagenomeSeq (v3.16)	GitHub	https://github.com/HCBravoLab/metagenomeSeq
Vegan (v2.6–2) & Phyloseq (v1.40.0)	McMurdie, Dixon et al.^10^^,^^11^	https://cran.r-project.org/web/packages/vegan/vegan.pdf & https://joey711.github.io/phyloseq/tutorials-index.html
DESeq2 (v1.38.3)	Love et al.^12^	https://www.bioconductor.org/packages/release/bioc/html/DESeq2.html
clusterProfiler (v4.8.3)	Guangchuang et al.^13^	https://bioconductor.org/packages/release/bioc/html/clusterProfiler.html
ggplot2 (v3.4.2)	CRAN	https://cran.r-project.org/web/packages/ggplot2/index.html

**Others**		

KEGG	Kanehisa and Goto^14^	https://www.genome.jp/kegg/
Centrifuge	Eppendorf 5810R refrigerated centrifuge	Cat. No. 5810R
ROTOSPIN rotary mixer	Tarsons	Cat. No. 3090X
Magnetic stand-96	Invitrogen	Cat. No. 12027
NanoDrop 2000 spectrophotometer	Thermo Fisher Scientific	Cat. No. ND2000CLAPTOP
Qubit 4.0 fluorometer	Thermo Fisher Scientific	Cat. No. Q33240
MinION Mk1C	Oxford Nanopore Technologies	MIN-101C
Bioanalyzer 2100 instrument	Agilent Technologies	Cat. No. G2939BA
NextSeq 2000 system	Illumina	Cat. No. 20038897

## Materials and equipment

All resources, materials, and software are listed in the key resources table.

### Bioinformatics analysis

All bioinformatics analyses have been carried out on the CSIR-IGIB’s high-performance GPU-linux based scientific computing server (CentOS 7.7) and on a local workstation. A basic knowledge of scripting languages (bash, R, and Python) is required to understand and apply this protocol. Software and scripts used in this protocol are provided in the “Software and algorithms” section of the key resources table.

### Computational requirements for analysis

Basic installation requirements:

• Memory = 128 GB.
• Cores = 4.
• compute nodes = 40.
• Operating system (Linux or Mac).
• GPUs (V100)-Linux-based (CentOS 7.7) compute cluster.

***Note:*** A computer with a linux and network connection is required. The RAM requirement depends on the number of samples to be analyzed. 16 GB RAM should be sufficient for an initial analysis. On the basis of availability of RAM, analysis time may fluctuate (fast).

## Step-by-step method details

### RNA isolation

**Timing: 2 h**

This major step describes the isolation of RNA from the nasopharyngeal swab samples collected from hospital admitted COVID-19 positive patients. RNA is extracted using commercially available RNA extraction kits (QIAmp viral mini kit, Qiagen, Cat. No. 52906), in accordance with the kit protocol (QIAamp Viral RNA Mini Handbook).

1. Lyse the sample (VTM).
  a. Take 200 μL of VTM solution in a 1.5 mL microcentrifuge tube.
  b. Add 560 μL prepared Buffer AVL containing carrier RNA.
  c. Mix thoroughly by pulse-vortexing for 15 s to ensure efficient lysis.
  d. Incubate at room temperature for 10 min.

**CRITICAL:** Buffer AVL–carrier RNA should be prepared fresh, and is stable at 2°C–8°C for up to 48 hours. This solution develops a precipitate when stored at 2°C–8°C that must be re-dissolved by warming at 80°C before use.

***Note:*** Add Buffer AVE to the tube containing lyophilized carrier RNA to obtain a solution of 1 μg/μL (i.e., add 310 μL Buffer AVE to 310 μg lyophilized carrier RNA). Dissolve the carrier RNA thoroughly, divide it into conveniently sized aliquots, and store it at −20°C. Do not freeze–thaw the aliquots of carrier RNA more than 3 times.

2. Binding of the viral RNA to the QIAamp membrane.
  a. Add 560 μL 96% ethanol to the sample.
  b. Mix by pulse-vortexing for 15 s.
  c. Carefully apply 630 μL of the solution to the QIAamp Mini column.
  d. Close the cap, and centrifuge at 6000 × *g* (8000 rpm) for 1 min.
  e. Place the QIAamp Mini column into a clean 2 mL collection tube, and discard the tube containing the filtrate.
  f. Repeat this step until all of the lysate has been loaded onto the spin column.

**CRITICAL:** Use only ethanol, since other alcohols may result in reduced RNA yield and purity. Only use freshly prepared ethanol.

3. Wash.
  a. Carefully open the QIAamp Mini column, and add 500 μL Buffer AW1.
  b. Close the cap and centrifuge at 6000 × *g* (8000 rpm) for 1 min.
  c. Place the QIAamp Mini column in a clean 2 mL collection tube and discard the tube containing the filtrate.
  d. Carefully open the QIAamp Mini column and add 500 μL Buffer AW2.
  e. Close the cap and centrifuge at full speed (20,000 × *g*; 14,000 rpm) for 3 min.

***Note:*** Buffer AW1 and AW2 are supplied as a concentrate. Before using for the first time, add the appropriate amount of ethanol (96%–100%) to Buffer concentrates.

4. Dry Spin.
  a. Place the QIAamp Mini column in a new 2 mL collection tube, and discard the old collection tube with the filtrate.
  b. Centrifuge at full speed for 1 min.

**CRITICAL:** Dry spin is always recommended since residual Buffer AW2 in the eluate may cause problems in downstream applications.

5. Elute.
  a. Place the QIAamp Mini column in a clean 1.5 mL microcentrifuge tube. Discard the old collection tube containing the filtrate.
  b. Carefully open the QIAamp Mini column and add 60 μL Buffer AVE equilibrated to room temperature.
  c. Close the cap and incubate at room temperature for 1 min.
  d. Centrifuge at 6000 × *g* (8000 rpm) for 1 min.
6. Quantify the isolated RNA using a NanoDrop Spectrophotometer taking 1 μL of the sample.
7. Store the viral RNA at −20°C or −80°C for long term storage.

**Pause point:** RNA collected can be stored at −80°C until library preparation. However, RNA is prone to degradation and samples should be used as soon as possible. To avoid possible batch effects due to different storage times, RNA from all study groups (Pre-VOCs, VOCs: Delta and Omicron) need to be included in each batch.

### Viral detection and quantification

**Timing: 2 h**

We perform a real-time reverse transcription polymerase chain reaction (RT-PCR) test for SARS-CoV-2 detection and quantification using TRUPCR SARS-CoV-2 kit (3B BlackBio Biotech India Ltd.).

8. Thaw the necessary kit (HiScribe T7 ARCA mRNA Kit (with tailing)) components, mix and pulse-spin in microfuge to collect solutions to the bottoms of tubes.
9. Reaction preparation.
  a. Prepare the reaction mix as follows:

**Table:** 

Reagent	For 1 rxn.	Storage
Master mix	10 μL	−20°C
Enzyme mix	0.35 μL	−20°C
Primer probe mix	4.65 μL	−20°C
**Total**	**15 μL**	**-**
  b. Transfer 15 μL of the above prepared Reaction mix into a 0.2 mL PCR tube.
  c. Add 10 μL of RNA sample, positive control, or negative control to make a final volume of 25 μL.
10. Program set up.
  a. Define the following setting for temperature profile.

**Table:** 

Stop	Temperature, °C	Time	Dye acquisition	Cycles
1	50	15 min	-	1
2	95	05 min	-	1
3	95	05 s	-	38
4	60	40 s	Yes	
5	72	15 s	-

***Note:*** Choose passive reference dye as “None”.

11. Channel selection.
  a. Define the following setting for channel selection.

**Table:** 

Detector name	Reporter	Quencher
E gene	Texas Red/Orange/ROX	None
RNase P	HEX/Yellow/VIC	None
RdRp + N gene	FAM/Green	None

12. Result analysis: We consider the cycle threshold (Ct) value of 35 for interpretation of the results.

### SARS-CoV-2 whole genome sequencing

**Timing: 16 h**

COVID-19 positive RNAs are subjected to sequencing, which allows the identification of SARS-CoV-2 variants for further classification of samples into Pre-VOCs and VOCs (Delta and Omicron).

***Note:*** Prepare the sequencing library following the recommendation of Oxford Nanopore Native Barcoding protocol.

13. Reverse transcription.
  a. Proceed with first strand cDNA generation from the isolated RNA using the following reaction mix.

**Table:** 

Reagent	Volume/well	Storage
RNA sample (∼100 ng)	11 μL	−20°C
60 μL random hexamers and anchored polyT(23)	1 μL	−20°C
10 mM dNTPs	1 μL	−20°C
**Total**	**13 μL**	**-**
  b. Do pipette mixing and give a short spin.
  c. Put the reaction plate for incubation at 65°C for 5 min, and then snap cool on ice for 1 min followed by first strand cDNA synthesis.
14. First strand cDNA synthesis.
  a. Add the following reagents to the annealed template RNA.

**Table:** 

Reagent	Volume/well	Storage
5X SuperScript IV buffer	4 μL	−20°C
100 mM DTT	1 μL	−20°C
RNaseOUT RNase inhibitor	1 μL	−20°C
SuperScript IV Reverse Transcriptase	1 μL	−20°C
**Total**	**7 μL**	**-**
  b. Mix using pipette and spin down the residual volume.
  c. Put the reaction plate in the thermal cycler for incubation as per using the below program.

**Table:** 

Steps	Temperature	Time	Cycles
	23°C	10 min	1
	50°C	10 min	1
	80°C	10 min	1
Hold	4°C	forever
  d. To degrade the RNA strand attached to the cDNA, add 1 μL of RNase H to each well.
  e. Mix properly and incubate at 37°C for 20 min.
15. Second strand cDNA synthesis.
  a. Prepare the following master mix and add it to cDNA.

**Table:** 

Reagent	Volume/well	Storage
cDNA	21 μL	−20°C
Random primers	1 μL	−20°C
10 mM dNTPs	1 μL	−20°C
10X Klenow Buffer	1 μL	−20°C
**Total**	**25 μL**	**-**
  b. Mix by vortexing, spin down the residual volume.
  c. Incubate the plate at 95°C for 3 min and snap cooled on ice for >1 min.
  d. Add 1 μL of Klenow Fragment to each well, followed by vortexing and spinning down the residual volume.
  e. Incubate the plate in a thermal cycler as per the program below.

**Table:** 

Steps	Temperature	Time	Cycles
	37°C	60 min	1
	75°C	10 min	1
Hold	4°C	forever
16. Double stranded cDNA purification.
  a. Bead Addition and Mixing: Add 45 μL of AMPure XP beads to the double-stranded cDNA and ensure thorough mixing by vortexing.
  b. Incubation: Incubate the mixture at room temperature for 15 min to allow binding of cDNA to the beads.
  c. Magnetic Separation: Place the plate on a magnetic stand for 5 min, allowing the beads to settle on the magnet’s side. Carefully remove the clear supernatant without disturbing the beads.
  d. Ethanol Wash: Wash the bead-bound cDNA twice with 100 μL of 80% ethanol to remove impurities. Ensure that residual ethanol is removed by spinning the plate.
  e. Air Drying: Allow the plate to air dry, ensuring that all residual ethanol evaporates.
  f. Resuspension: Resuspend the bead-bound cDNA in 26 μL of resuspension buffer and incubate for 2 min at room temperature.
  g. Elution: Carefully elute 25 μL of purified double-stranded cDNA from the beads.
  h. Concentration Measurement: Measure the concentration of the purified cDNA product using a NanoDrop or similar instrument.
17. PCR tiling of SARS-CoV-2 virus.
  We amplify the SARS-CoV-2 genome from the double-stranded cDNA using ARTIC V3 primers specific for SARS-CoV-2.
  ***Note:*** Divide these primers into two pools: Pool A and Pool B, and dilute to a concentration of 10 nM before being utilized in the subsequent step.
  a. Prepare the reaction mixture according to the following instructions.
    **Table:** PCR reaction master mix

Reagent	Volume (pool A)	Volume (pool B)	Storage
Purified double stranded cDNA (∼100 ng)	x μl	x μl	−20°C
Q5 Hot Start High-Fidelity 2X Master Mix	12.5 μL	12.5 μL	−20°C
V2 Primer pool at 10 μM (A or B)	3.7 μL	3.7 μL	−20°C
V3 Primer pool at 10 μM (A or B)	0.45 μL	0.38 μL	−20°C
Nuclease-free water	8.35-x μl	8.42-x μl	RT
**Total**	**25 μL**	**25 μL**	**-**
  b. Gently mix the reaction plate by pipetting and incubate in a thermal cycler as per the program below.
    **Table:** PCR cycling conditions

Steps	Temperature	Time	Cycles
Initial Denaturation	98°C	30 s	1
Denaturation	98°C	15 s	35 cycles
Annealing and Extension	65°C	5 min	
Hold	4°C	forever
  c. Briefly spin the plate after PCR reaction and perform the following steps:
    i. Centrifugation: Centrifuge the plate once before proceeding to the purification step.
    ii. Combining Pools: Combine the reactions from Pool A and Pool B into a new deep 96-well plate, with one well designated for each sample. Then, add 50 μL of resuspended AMPure XP beads to each well.
    iii. Incubation: Incubate the plate at room temperature for 10 min.
    iv. Magnetic Separation: Subsequently, transfer the plate to a magnetic stand and wait for 5 min. Once the beads have completely moved to the side of the magnet, carefully discard the supernatant.
    v. Ethanol Wash: Wash the pellet with 200 μL of 80% ethanol twice to remove any impurities. Afterwards, remove the residual ethanol by spinning the plate, and allow it to air dry.
    vi. Elution: Elute the amplified product using 15 μL of nuclease-free water.
    vii. Dilution and Quantification: After diluting the eluted product in 1:10 ratio, quantify using the Qubit High Sensitivity dsDNA assay kit.
18. End-prep.
  In order to add the barcodes to the amplicons, short PolyA should be added to the end of the amplicons.
  a. Prepare the following reaction mix:

**Table:** 

Reagent	Volume	Storage
cDNA (∼200 ng)	x μL	−20°C
Nuclease-free water	(12.5 - x) μL	−20°C
Ultra II End-prep reaction buffer	1.75 μL	−20°C
Ultra II End-prep enzyme mix	0.75 μL	−20°C
**Total**	**15 μL**	**-**
  b. Gently mix the reaction by pipetting up and down 8–10 times and spin down the plate.
  c. Incubate at 20°C for 5 min and 65°C for 5 min in a thermal cycler.
  d. Spin the plate.
19. Native barcode ligation.
  To the end-prepped DNA, unique barcode sequences are added.
  a. Spin down the Native Barcode plate in a microplate centrifuge.
  b. Based on the number of samples chosen for sequencing, prepare the following reaction mix:

**Table:** 

Reagent	Volume	Storage
End-prepped DNA	5.5 μL	−20°C
Nuclease-free water	1.5 μL	RT
Native Barcode	2.5 μL	−20°C
NEBNext Ultra II Ligation Master Mix	10 μL	−20°C
NEBNext Ligation Enhancer	0.5 μL	−20°C
**Total**	**15 μL**	**-**
  c. Gently mix the reaction by pipetting and incubate at 20°C for 20 min and at 65°C for 10 min in a thermal cycler.
  d. Spin down the plate.
20. Pooling Barcoded Samples: Combine the barcoded samples into one tube. Add 8 μL of AMPure XP beads to the tube. Incubate at room temperature for 10 min on a hula mixer.
21. Magnetic Separation: Transfer the tube to a magnetic stand for 5 min. Carefully remove the clear supernatant once the beads have moved towards the magnet completely.
22. Washing: Resuspend the pellet in 700 μL of Short Fragment Buffer (SFB). Return the tube to the magnet, and remove the supernatant. Repeat this step for a total of two washes.
23. Ethanol Wash: Wash the pellet with 100 μL of 80% ethanol.
24. Elution: Elute the pooled barcoded sample with 35 μL of nuclease-free water.
25. Quantification: Quantify the pooled barcoded sample using the Qubit High Sensitivity dsDNA assay kit.
26. Add the sequencing adapter to the pooled barcoded sample as per the following reaction:

**Table:** 

Reagent	Volume	Storage
End-prepped DNA	5.5 μL	−20°C
Adapter Mix II (AMII)	5 μL	−20°C
NEBNext Quick Ligation Reaction Buffer (5X)	10 μL	−20°C
Quick T4 DNA Ligase	5 μL	−20°C
**Total**	**15 μL**	**-**
  a. Gently pipette mix and spin the tube.
  b. Incubate the sample at room temperature for 20 min.
27. Purification: Purify the final library using 20 μL AMPure XP beads. Following separation of beads from the library by placing onto the magnetic stand, wash the pellet twice using 125 μL Short Fragment Buffer (SFB) and elute with 15 μL Elution Buffer.
28. Quantification: Quantify the final library using 1 μL of library in Qubit High sensitivity dsDNA assay kit.
  a. If concentration is above 100 ng/μL, dilute with EB buffer for a final concentration of 50 ng/μL in a volume of 13 μL. Measure the concentration again using 1 μL of sample in Qubit.
29. Priming and loading the SpotON flow cell.
  a. Allow the flow cell to come to room temperature (∼ 24°C) for at least 5 min before inserting it into the MinION Mk1B device.
  b. Determine the number of active pores in the flow cell by selecting “Check flow cell” in MinKNOW.
  c. Open the priming port and check for a small air bubble under the cover.
    i. Draw back a small volume to remove any bubble:
    ii. Insert a P1000 tip into the priming port.
    iii. Turn the pipette’s thumbwheel anti-clockwise until the dial shows a gain of no more than 20–30 μL or until a small volume of storage buffer (yellow color) is entering the pipette tip.
  d. Prepare the flow cell priming mix by adding 30 μL of FLT to the entire tube (1,170 μL) of FB, and mix by pipetting, being careful to avoid foaming/air bubbles.
  e. The priming process involved two steps:
    i. First Priming -
      Open the priming port.
      Slowly, load a total of 800 μL of the priming solution into the flow cell.
      Allow the flow cell to incubate at room temperature for 5 min.
    ii. Second Priming:
      For the second priming step, open the SpotON port as well.
      Add 200 μL of priming solution into the flow cell.
      These steps ensure that the MinION flow cell is properly primed and ready for sequencing.
30. Sequencing run.
  a. The final library was prepared for loading as per the formula mentioned below.

**Table:** 

Reagent	Volume	Storage
Sequencing Buffer (SQB)	37.5 μL	−20°C
Loading Beads (LB), mixed immediately before use	25.5 μL	−20°C
DNA library (100 ng)	12 μL	−20°C
**Total**	**75 μL**	**-**
  b. Mix the entire library very slowly by pipetting, followed by loading into the flow cell through the SpotON port in a dropwise manner.
  c. Configure the MinION Mk1c software for sequencing, selecting the high-accuracy base calling option. This configuration was done after providing the relevant information about the library preparation kit, including SQK-LSK109, EXP-NBD104, and EXP-NBD114.
  d. Allow the sequencing process to continue until a sufficient amount of data has been generated, and subsequently, stop the sequencing run.
  e. Once the sequencing run has been stopped, wash the flow cell using a Flow Cell wash kit, and QC it to confirm if there are still a sufficient number of functional pores on the flow cell for another sequencing run.
  f. Following this, store the flow cell at 4°C to preserve its integrity, and transfer the generated sequencing data to an HPC cluster for further data analysis.

### Data analysis and interpretation for SARS-CoV-2 genome sequencing

**Timing: 5–6 h**

This step explains the detailed description of the analysis methods used for interpretation of the SARS-CoV-2 variant.

31. Data transfer from nanopore sequencing device (Mk1B/Mk1C).
  a. The data is stored in the device’s “/data/” folder with the run name.
  b. Transfer the data using “scp” (relatively slow) or “rsync” (fast copying) tool.

***Note:*** It can copy locally, to/from another host over any remote shell, or to/from a remote rsync daemon.

32. Software Setup for SARS-CoV-2 genome analysis.
  a. Operating System Requirement: A 64-bit UNIX, Linux, or a similar environment. This includes Mac OS, Linux distributions such as Ubuntu 16 or later, or Windows 10 Subsystem for Linux.
  b. Create a custom conda environment: Install the relevant version of Miniconda (recommended as it is relatively small and quick to install) following the installation guidelines at www.conda.io/projects/conda/en/latest/user-guide/install/index.html.
  c. Install ARTIC nCoV-2019 specific data and software repository: Update the environment using the following command.

git clone https://github.com/artic-network/artic-ncov2019.git

cd artic-ncov2019

conda env remove -n artic-ncov2019

conda env create -f environment.yml

33. Make a new directory for analysis: Give a meaningful name to the analysis directory e.g., analysis/run_name.

mkdir analysis

cd analysis

mkdir run_name

cd run_name

mkdir basecalling demultiplexing guppyplex nanopolish

34. Activate the ARTIC environment: Perform all steps in this tutorial in the artic-ncov2019 conda environment:

source activate artic-ncov2019.

35. Basecalling with Guppy:

guppy_basecaller -c dna_r9.4.1_450bps_fast.cfg -i /path/to/Runfolder/ -s ./basecalling/ -x "cuda:1" -r.

**Figure:**
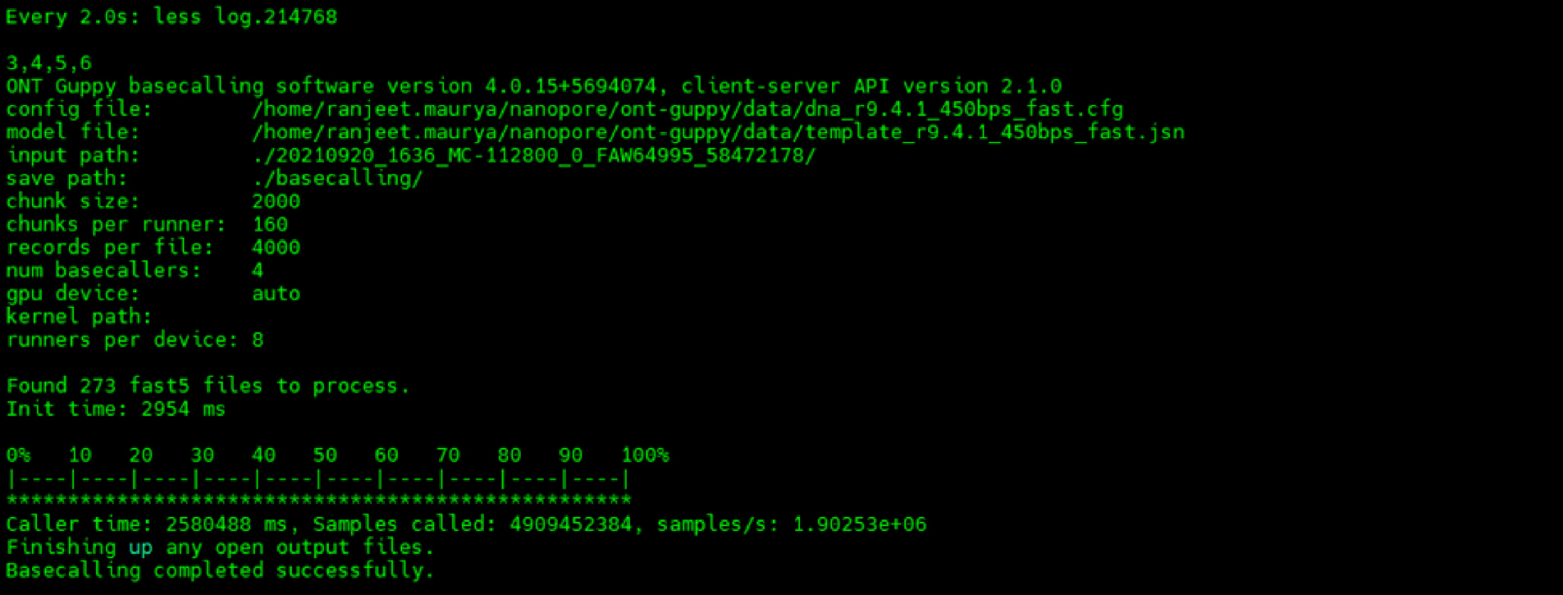

36. Demultiplexing with Guppy:

guppy_barcoder --require_barcodes_both_ends -i ./basecalling/ -s ./demultiplexing/ --arrangements_files "barcode_arrs_nb12.cfg barcode_arrs_nb24.cfg" -x "cuda:1"

**Figure:**
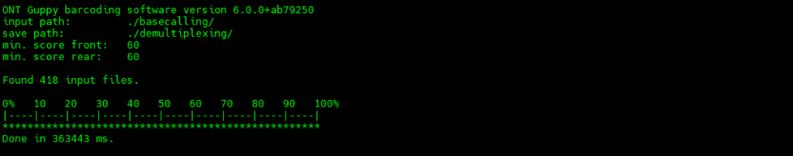

37. Read filtering: Perform this step for individual barcodes.

cd guppyplex

artic guppyplex --skip-quality-check –directory ../demultiplexing/barcode01.

38. Run the ARTIC MinION pipeline: Perform this step for individual barcodes.

artic minion --normalise 200 --scheme-directory /home/ranjeet/nanopore/artic-ncov2019/primer_schemes/ --read-file ../guppyplex/barcode01.fastq --fast5-directory ../path/to/Sequencing_run/fast5_files/ --sequencing-summary ../path/to/sequencing_summary.txt nCoV-2019/V3 Sample_name

#Where,

--scheme-directory: specifies the directory containing primer schemes for the analysis.

--read-file: specifies the location of the input FASTQ file containing the sequencing reads.

--fast5-directory: specifies the directory containing the raw FAST5 files generated by the sequencer.

--sequencing-summary: specifies the location of the sequencing summary file. This file contains information about the sequencing run, such as the number of reads generated, quality metrics.

“nCoV-2019/V3”: specifies the primer scheme specific to the novel coronavirus(nCoV-2019) targeting version 3 of the genome.

“Sample_name”: specifies the name of the sample being analyzed.

Replace /path/to/ with the actual paths to the relevant directories and files on system.

***Note:*** In the above code, “--normalise 200” parameter is used to normalize coverage depth to 200X during data analysis. This adjustment aims to enhance the robustness of results by ensuring a more consistent coverage across the sequenced regions.

**Table:** Output files

File name	Description
samplename.rg.primertrimmed.bam	BAM file for visualisation after primer-binding site trimming
samplename.trimmed.bam	BAM file with the primers left on (used in variant calling)
samplename.merged.vcf	all detected variants in VCF format
samplename.pass.vcf	detected variants in VCF format passing quality filter
samplename.fail.vcf	detected variants in VCF format failing quality filter
samplename.primers.vcf	detected variants falling in primer-binding regions
samplename.consensus.fasta	consensus sequence

39. Merge the Variant call files: Merge multiple variant call format (vcf) files into a single VCF file.

bcftools merge --force-samples ∗.vcf.gz > all_sample.vcf.

40. Convert the barcode name with Sample name: Perform this step for individual barcodes.

for filename in barcode01∗; do echo mv ∖"$filename∖" ∖"${filename//barcode01/IGIB1130001}∖"; done | /bin/bash.

# Here, “| /bin/bash/” pipes the printed 'mv' commands into the Bash shell (/bin/bash) for execution.

# An alternative approach using 'rename' command for converting barcode names to sample names for each sample. rename -v 's/barcode01/IGIB1130001/' ∗

41. Change of SARS-CoV-2 header with Institute tag name for all the samples

for i in ∗.fasta;do temp = ${i%.∗};echo "sed '1s/>MN908947.3/>${temp}_CSIR-IGIB/g' $i > ./fastachanged/$temp.fasta";done > name.sh

bash name.sh.

# An alternative way to change of SARS-CoV-2 header with sample tag name for each samples individually.

sed '1s/>MN908947.3/>${temp}_CSIR-IGIB/g' barcode01.fasta > ./fastachanged/barcode01.consensus.fasta

sed '1s/>MN908947.3/>${temp}_CSIR-IGIB/g' barcode02.fasta > ./fastachanged/barcode02.consensus.fasta

sed '1s/>MN908947.3/>${temp}_CSIR-IGIB/g' barcode03.fasta > ./fastachanged/barcode03.consensus.fasta.

42. Concatenate all fasta to one file.

cat ∗.consensus.fasta > concatenated_consensus.fasta.

**Figure 1:**
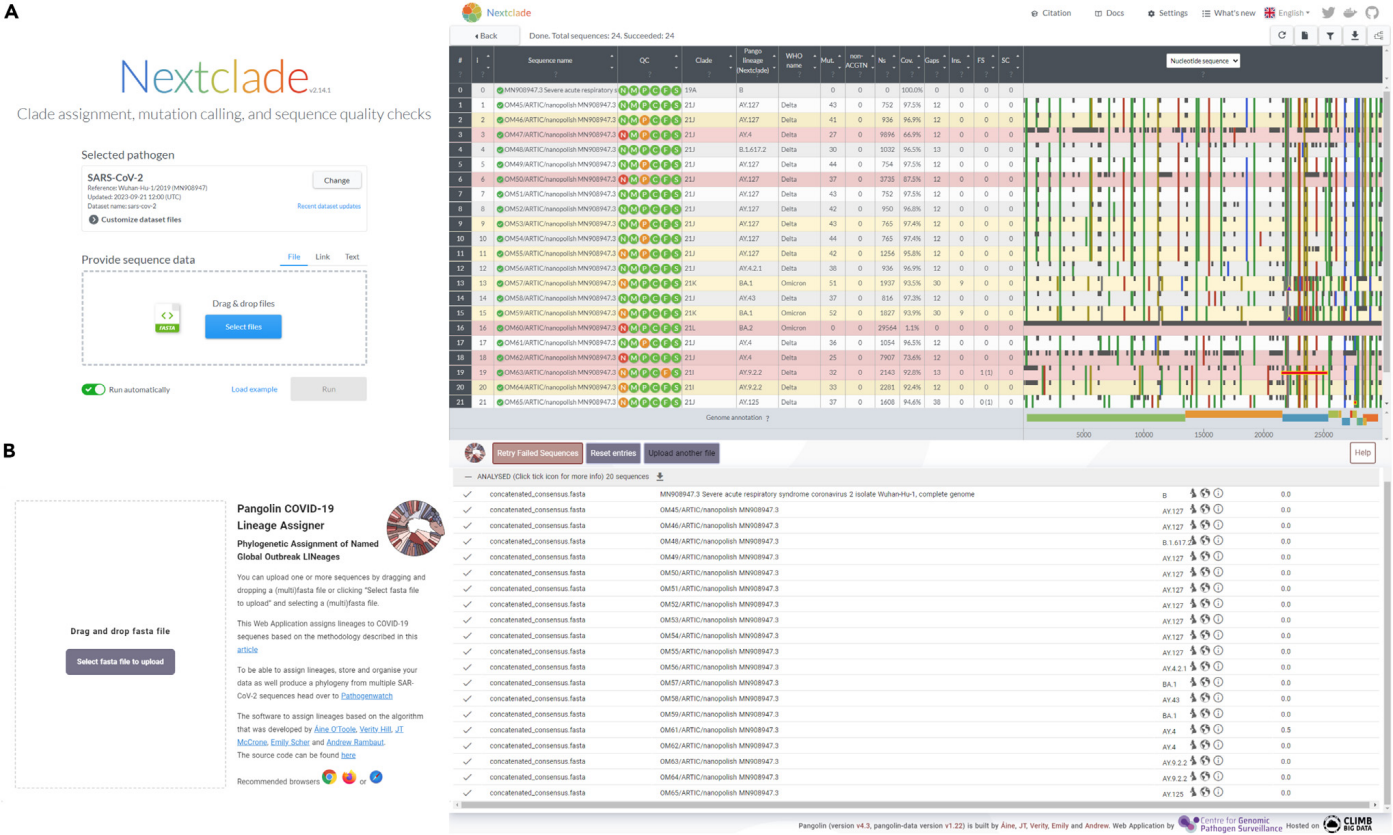
Clade and Lineage classification (A) Nextclade webtool for clade and (B) Pangolin webtool for lineage classification.

43. Upload the concatenated fasta files to the Nextclade and Pangolin webtool for clade and lineage classification respectively (Figure 1).

### RNA-seq library preparation

**Timing: 13–14 h**

**Timing: 15 min (for step 44)**

**Timing: 1 h 30 min (for step 45)**

**Timing: ∼ 50 min with handling time (for step 46)**

**Timing: ∼ 2 h with handling time (for step 47)**

**Timing: ∼ 45 min with handling time (for step 48)**

**Timing: ∼2 h 30 min with handling time (for step 49)**

**Timing: ∼2 h with handling time (for step 50)**

**Timing: 1 h 30 min (for step 51)**

**Timing: 1 h (for step 52)**

This method explains how to convert total RNA into a library suitable for subsequent sequencing. Steps involve RNA quantification, ribosomal RNA removal, RNA fragmentation, first and second cDNA strand synthesis, 3′ end adenylation, adapter ligation, DNA fragment enrichment, final library quality and quantity assessment, cluster generation, and sequencing of reads 1 and 2.

44. RNA Quality Check.
  a. Check the concentration and purity of the isolated RNA using the Nanodrop system.
  b. For library preparation, take the input RNA volume according to the 250 ng concentration and make up the volume to 10 μL using nuclease-free water.
45. Depletion of rRNA from total RNA.
  In this step, rRNA is depleted from each sample and further purified, fragmented and primed for cDNA synthesis.
  Bind rRNA
  a. In a 96-well plate, add an input concentration of 250 ng of the RNA and make up the volume to 10 μL for each sample using NFW.
  b. Add 5 μL RBB (rRNA Binding Buffer) to each well.
  c. Add 5 μL RRM G (rRNA Removal Mix - Gold) specific for the Ribo-zero gold kit to the well.
  d. Carefully mix the reagents by pipetting up and down 10 times (Do not vortex mix).
  e. Centrifuge at 280 *g* for 1 min.
  f. Incubate the reaction plate in the thermal cycler at 68°C for 5 min to allow denaturation of the RNA.
  g. Once the reaction is over, take the plate out and incubate at room temperature for 1 min.
    Remove rRNA
  h. Thoroughly vortex and add 35 μL RRB (rRNA Removal Beads) to each well of the above plate.
  i. Again, mix by pipetting up and down 10 times.
  j. Incubate at room temperature for 1 min.
  k. Place the plate on a magnetic stand for 1 min.
  l. Transfer all the supernatant to the corresponding well of the 96-well plate.
    Clean up RNA
  m. Vortex RNAClean XP Beads until well dispersed.
  n. Add 99 μL or 193 μL (if starting with degraded total RNA) RNAClean XP Beads to each well, and then mix thoroughly by pipetting up and down nearly 10 times.
  o. Incubate at room temperature for 15 min.
  p. Place on a magnetic stand and wait until the liquid is clear (∼5 min).
  q. Remove and discard all of the supernatant from each well.
  r. Ethanol washing:
    i. Add 200 μL freshly prepared 70% EtOH to each well to wash.
    ii. Incubate on the magnetic stand for 30 s.
    iii. Remove and discard all supernatant from each well. (Remove all the residual EtOH from each well).
    iv. Air-dry on the magnetic stand for 15 min.
    v. Remove from the magnetic stand.
      ***Note:*** Always use freshly prepared Ethanol. Make sure all the drops of ethanol are removed/ dried from the walls of the plate before adding ELB.
  s. Elution:
    i. Add 11 μL ELB to each well, and then mix thoroughly by pipetting up and down.
    ii. Incubate at room temperature for 2 min.
    iii. Centrifuge at 280 × *g* for 1 min.
    iv. Place on a magnetic stand and wait until the liquid is clear (∼5 min).
    v. Transfer 8.5 μL supernatant to the corresponding well.
  t. Add 8.5 μL EPH to each well, and then mix thoroughly by pipetting up and down.
  u. Incubate on the thermal cycler at 94°C for 8 min and hold at 4°C.
  v. Centrifuge briefly after taking out of the thermal cycler.
    **CRITICAL:** Always avoid mixing using a vortexer during the complete library preparation protocol (do pipette mixing). All the beads must be taken out and let stand at room temperature for 30 min (never use cold beads). Especially, rRNA Removal Beads are highly dense, requiring meticulous and deliberate pipetting for precise and accurate handling. During cleanup, transfer the supernatant carefully by checking under a white light or on a white surface for the beads on tips.
46. First strand cDNA synthesis.
  In this step, the cleaved RNA fragments are reverse transcribed into the first strand cDNA.
  Check the concentration and purity of the isolated RNA using the Nanodrop system.
  a. Add SuperScript IV at a ratio of 1 μL SuperScript IV to 9 μL FSA.
    ***Note:*** SuperScript IV is an enzyme and should be taken out from −20°C temperature at the time of use only, and in a cooler/ ice. FSA should be handled with precautions since it contains Actinomycin D, a toxin
  b. Add 8 μL FSA and SuperScript II mixture to each well of the plate, and then mix thoroughly by pipetting up and down.
  c. Centrifuge at 280 × *g* for 1 min.
  d. Incubate on the preprogrammed thermal cycler and run the following “synthesize 1st Strand program”. Each well contains 25 μL.

**Table:** 

Steps	Temperature	Time	Cycles
1^st^ strand synthesis	25°C	10 min	1
	42°C	15 min	1
	70°C	15 min	1
Hold	4°C	forever
47. Second strand cDNA synthesis.
  This process removes the RNA template, synthesizes a replacement strand, and incorporates dUTP in place of dTTP to generate ds cDNA. The incorporation of dUTP quenches the second strand during amplification. Magnetic beads separate the ds cDNA from the second strand reaction mix. The result is blunt-ended cDNA.
  a. Dilute CTE to 1:50 in RSB. For example, 2 μL CTE + 98 μL RSB.
  b. Add 5 μL diluted CTE to each well. Discard diluted CTE after use.
  c. Add 5 μL RSB to each well.
  d. Centrifuge SMM at 600 × *g* for 5 s.
  e. Add 20 μL SMM to each well, and then mix thoroughly by pipetting up and down 6 times.
  f. Centrifuge at 280 × *g* for 1 min.
  g. Place on the preprogrammed thermal cycler and incubate at 16°C for 1 h. Each well contains 50 μL.
  h. Place on the bench and let stand to bring to room temperature.
  i. Purify cDNA.
    i. Add 90 μL AMPure XP beads to each well of the DFP plate.
    ii. Mix thoroughly by pipetting up and down 10 times.
    iii. Incubate at room temperature for 15 min.
    iv. Centrifuge at 280 × *g* for 1 min.
    v. Place on a magnetic stand and wait until the liquid is clear (∼5 min).
    vi. Remove and discard 135 μL supernatant from each well.
  j. Two times ethanol wash.
    i. Add 200 μL fresh 80% EtOH to each well.
    ii. Incubate on the magnetic stand for 30 s.
    iii. Remove and discard all supernatant from each well.
    iv. Use a 20 μL pipette to remove residual EtOH from each well.
    v. Air-dry on the magnetic stand for 15 min. Do not over dry beads.
  k. Elution.
    i. Remove from the magnetic stand.
    ii. Add 17.5 μL RSB to each well, and then pipette mix thoroughly.
    iii. Incubate at room temperature for 2 min.
    iv. Centrifuge at 280 × *g* for 1 min.
    v. Place on a magnetic stand and wait until the liquid is clear (∼5 min).
    vi. Transfer 15 μL supernatant to the corresponding well of the plate.
      **Pause point:** If you are stopping, seal the plate and store at −25°C to −15°C for up to 7 days.
48. Adenylate 3′Ends.
  One adenine (A) nucleotide is added to the 3ʹ ends of the blunt fragments to prevent them from ligating to each other during adapter ligation reaction. One corresponding thymine (T) nucleotide on the 3ʹ end of the adapter provides a complementary overhang for ligating the adapter to the fragment. This strategy ensures a low rate of chimera (concatenated template) formation.
  a. Dilute CTA to 1:100 in RSB. For example, 1 μL CTA + 99 μL RSB.
  b. Add 2.5 μL diluted CTA to each well. Discard diluted CTA after use.
  c. Centrifuge ATL at 600 × *g* for 5 s.
  d. Add 12.5 μL ATL to each well, and then mix thoroughly by pipetting up and down 10 times.
  e. Seal the ALP plate with a Microseal 'B' adhesive seal.
  f. Centrifuge at 280 × *g* for 1 min.
  g. Put the samples on the thermal cycler and initiate the ATAIL70 program. Utilize the preheat lid option, setting it to 100°C, and then proceed with incubation at 37°C for 30 min, followed by 70°C for 5 min. Hold at 4°C. Each well contains 30 μL.
  h. Centrifuge at 280 × *g* for 1 min.
49. Ligate Adapters.
  This process ligates multiple indexing adapters to the ends of the ds cDNA fragments, which prepares them for hybridization onto a flow cell.
  a. Dilute CTL 1:100 in RSB. For example, 1 μL CTL + 99 μL RSB. Discard the diluted CTL after use.
  b. Remove LIG from −25°C to −15°C storage.
  c. Add the following reagents in the order listed to each well.
    i. Diluted CTL (2.5 μL).
    ii. LIG (2.5 μL).
    iii. RNA adapters (2.5 μL).
  d. Mix thoroughly by pipetting up and down 10 times.
  e. Centrifuge at 280 × *g* for 1 min.
  f. Incubate at 30°C for 10 min on the thermal cycler.
  g. Add 5 μL STL to each well, and then pipette mix thoroughly.
  h. Centrifuge at 280 × *g* for 1 min.
  i. CleanUp Ligated Fragments: This cleanup is done in 2 rounds with different volumes of AMPure XP beads and RSB. Perform steps i. to xvii using the Round 1 volumes. Repeat steps i. through xvii with the new plate using the Round 2 volumes.

**Table:** 

Reagent	Round 1	Round 2
AMPure XP beads	42 μL	50 μL
RSB	52.5 μL	22.5 μL
    i. Add AMPure XP beads to each well.
    ii. Mix thoroughly by pipetting up and down.
    iii. Incubate at room temperature for 15 min.
    iv. Centrifuge at 280 × *g* for 1 min.
    v. Place on a magnetic stand and wait until the liquid is clear (2–5 min).
    vi. Remove and discard all supernatant from each well.
    vii. Wash two times with 200 μL fresh 80% EtOH by incubating on the magnetic stand for 30 s.
    viii. Remove and discard all supernatant from each well.
    ix. Air-dry on the magnetic stand for 15 min.
    x. Remove from the magnetic stand.
    xi. Add RSB to each well and mix by pipetting.
    xii. Incubate at room temperature for 2 min.
    xiii. Centrifuge at 280 × *g* for 1 min.
    xiv. Place on a magnetic stand and wait until the liquid is clear (2–5 min).
    xv. Transfer 50 μL supernatant to the corresponding well of the CAP plate.
    xvi. Perform round 2 clean up and transfer 20 μL supernatant to the corresponding well of the PCR plate.
      **Pause point:** If you are stopping, seal the plate and store at −25°C to −15°C for up to 7 days.
50. Enrich DNA Fragments.
  This process uses PCR to selectively enrich those DNA fragments that have adapter molecules on both ends and to amplify the amount of DNA in the library. PCR is performed with PPC (PCR Primer Cocktail) that anneals to the ends of the adapters. Minimize the number of PCR cycles to avoid skewing the representation of the library.
  a. Amplify DNA Fragments.
    i. Place the PCR plate on ice and add 5 μL PPC to each well.
    ii. Add 25 μL PMM to each well, and then mix thoroughly by pipetting up and down 10 times.
    iii. Centrifuge at 280 × *g* for 1 min.
    iv. Place on the preprogrammed thermal cycler and run the PCR program. Each well contains 50 μL.
    v. Choose the preheat lid option and set to 100°C.
      **Table:** PCR reaction master mix

Reagent	Amount
PPC	5 μL
PMM	25 μL
Purified library	20 μL
      **Table:** PCR cycling conditions

Steps	Temperature	Time	Cycles
Initial Denaturation	98°C	30 s	1
Denaturation	98°C	10 s	15 cycles
Annealing	60°C	30 s	
Extension	72°C	30 s	
Final extension	72°C	5 min	1
Hold	4°C	forever
  b. Clean Up Amplified DNA.
    i. Centrifuge at 280 × *g* for 1 min.
    ii. Add 47.5 μL AMPure XP beads to each well. Adapter Type Volume AMPure XP beads.
    iii. Mix thoroughly by pipetting up and down 10 times.
    iv. Incubate at room temperature for 15 min.
    v. Centrifuge at 280 × *g* for 1 min.
    vi. Place on a magnetic stand and wait until the liquid is clear (2–5 min).
    vii. Remove and discard all supernatant from each well.
    viii. Wash two times with 200 μL fresh 80% EtOH as follows.
    ix. Incubate on the magnetic stand for 30 s.
    x. Remove and discard all supernatant from each well.
    xi. Air-dry on the magnetic stand for 15 min.
    xii. Remove from the magnetic stand and add 32.5 μL RSB to each well, and then mix thoroughly by pipetting up and down 10 times.
    xiii. Incubate at room temperature for 2 min.
    xiv. Centrifuge at 280 × *g* for 1 min.
    xv. Place on a magnetic stand and wait until the liquid is clear (2–5 min).
    xvi. Transfer 30 μL supernatant to the corresponding well of the TSP1 plate.
      **Pause point:** If you are stopping, seal the plate and store at −25°C to −15°C for up to 7 days.
51. Quantify Libraries.
  a. Use 1 μL sample to quantify all the libraries individually using the Qubit dsDNA HS Assay kit.
  b. Check the size of the quantified libraries using a DNA 1000 chip on an Agilent Technologies 2100 Bioanalyzer using Bioanalyzer High Sensitivity DNA kit.

**Figure 2:**
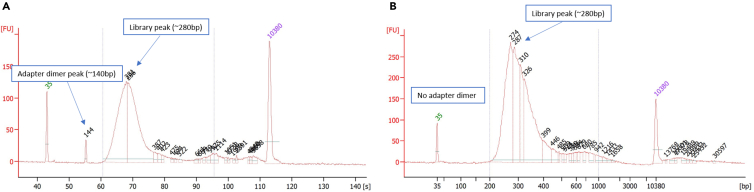
BioAnalyzer profile for the prepared library (A) 2 peaks indicating the adapter dimer (140 bp) and library (280 bp). (B) After cleanup with beads, adapter dimer removed and only 1 peak indicating purified library.

***Note:*** Expect the final product to be a band at ∼280 bp. A single peak should be observed for the library. If an unexpected small peak is observed at 120–170 bp, it indicates the presence of adapter dimers (Figure 2A). If adapter dimers are present in the library, perform an additional clean-up step with AMPure beads (second round of purification may reduce the library yields). A bead ratio of 0.8x to 1x is usually recommended and sufficient to remove the unwanted adapter dimers (Figure 2B).

**CRITICAL:** One day prior to anticipated run, remove cartridge from −20°C storage. Thaw at room temperature for 6 hours. Transfer to refrigerator 4°C, and continue to thaw for a minimum of 12 hours for 300 cycle or smaller kits. Before beginning with normalization and pooling, thaw the cartridge at room temperature, cartridge should have air on all sides except the bottom and should not be stacked. The NextSeq flow cell should also be taken out from 4°C at this point (half an hour before final loading of the library)

52. Normalize and Pool Libraries.
  ***Note:*** For best practice, perform normalization and pooling directly prior to sequencing. To minimize index hopping, do not store libraries in the pooled form.
  a. Calculate the concentration of the libraries in nM based on the ng/μL value using the formula: (concentration in ng/μL) / (660 *g*/mol × average library size) × 10ˆ6 = concentration in nM.
  b. Dilute the libraries to the concentration of 4 nM.
  c. Take 5 μL of each library to prepare a single pool for the total samples intended for the NextSeq run.
  d. Quantify the pooled cDNA library using Qubit for 4 nM.
  e. At this stage, do the following calculations to prepare a final concentration of 650 pmol.
53. Start sequencing.
  a. Mix the cartridge for sequencing on the NextSeq 2000 at 2 × 151 read length at a final loading concentration of 650 p.m.
  b. Insert the flow cell into the cartridge.
  c. Load 20 μL of the 650 pm library into the cartridge.
  d. Place the cartridge into the NextSeq 2000 instrument and start sequencing.

### Meta-transcriptome analysis

**Timing: 9–10 days (according to the availability of computational resources, may take less time)**

**Timing: 5 h (depending on the core availability might run in less time also) (for step 54)**

**Timing: ∼ 10 h (Trimmomatic) (for step 55)**

**Timing: ∼ 2 days (depending on the core availability might run in less time also) (for step 56)**

**Timing: ∼ 5 h (for step 57)**

**Timing: ∼ 5 h (for step 58)**

**Timing: ∼ 2 days (for steps 59–61)**

**Timing: ∼ 4 days (for step 62)**

Submit the following batch script to run analysis for quality check, removal of adapters and low quality sequences, alignment, taxonomic classification as well as functional analysis of microbes in parallel to the files where paired reads were concatenated into single files if needed.

54. Quality check of reads.
  a. Use FastQC to see the quality of each sample.
  b. Use the below code to run the samples.

fastqc -o out_file rawdata/file1_R1.fastq.gz

55. Remove adapter sequences.
  a. For each sample, filter and trim the raw metatranscriptomic reads using Trimmomatic v0.39.
  b. Batch script is given below to run the samples in parallel.
  c. Use the below code to run the analysis with precise input and output files.

#!/bin/bash.

##run: sbatch job_script.sh.

#SBATCH --job-name = pallawi_job

#SBATCH --nodes = 3 #squeue

#SBATCH --ntasks-per-node = 35.

#SBATCH --mem = 128 GB.

#Memory (RAM) per node. Number followed by unit prefix.

#SBATCH --partition = compute.

#Partition/queue in which run the job.

adapter = /home/pallawik/anaconda3/share/trimmomatic-0.39–2/adapters

rawdata = /lustre/pallawik/humann3/raw_data_voc_humann/raw_data

outdata = /lustre/pallawik/humann3/raw_data_voc_humann/Analysis

for i in $(ls ∗.fastq.gz |rev | cut -c 13- | rev | uniq)

do.

#remove adapter sequences

trimmomatic PE -threads 20 $rawdata/${i}_R1.fastq.gz $rawdata/${i}_R2.fastq.gz $trim_out/paired2/${i}_R1.fastq.gz $trim_out/unpaired2/${i}up_R1.fastq.gz $trim_out/paired2/${i}_R2.fastq.gz $trim_out/unpaired2/${i}up_R2.fastq.gz ILLUMINACLIP:$adapter/TruSeq3-SE.fa:2:30:10 LEADING:3 TRAILING:3 SLIDINGWINDOW:4:15 MINLEN:36

done.

56. Mapping of human RNA reads to the human reference sequence.
  Time (Index build): ∼15–20 min.
  a. Download reference sequence using the following link here.
  b. Install HISAT2 as given in “installation of tools/softwares/packages before analysis” section.
  c. Build index for human reference sequence using the code given below.
  d. Map the raw reads using the HISAT2 v2.2.1 algorithm onto the human reference.
    # Here, the command “hisat2-build” is used to create index of human reference sequence.
    hisat2-build -p 16 genome-human.fa genome-human.
    # hisat2 was used to map the query sequence from human reference sequence. Since, hisat gives output as .sam file, samtools is used to sort and convert the output in .bam file using the code below.
    hisat2 -p 100 --mp 3 --rdg 3 -x $index_hisat −1 $rawdata/${i}_R1.fastq.gz −2 $rawdata/${i}_R2.fastq.gz --summary-file $outdata/hisat2/smry/${i}.txt | $samtools view -bSu - | $samtools sort -o $outdata/hisat2/h2_out/${i}.bam.
    ***Note:*** The parameter values as given to “--mp” (mismatch penalty) and “--rdg” (read-gap penalty) are taken based on recommendation of the published paper by Kim et al. https://doi.org/10.1038/s41587-019-0201-4.
  e. Result HISAT2 analysis (HISAT Output).

**Figure:**
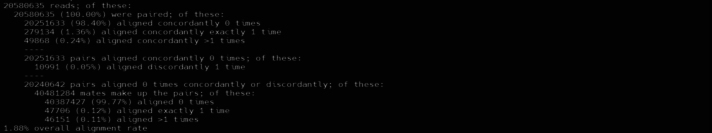
57. Identification and removal of human RNA reads from meta-transcriptomic (Unmapped) data.
  This step removes the aligned human RNA reads.
  a. Use Samtools to generate qualified non-human RNA-seq data.
  b. Rest unaligned reads are considered as microbial and used in taxonomic classification.
    samtools view -@ 50 -b -f 2 $outdata/${i}.bam > mapped/${i}_mapped.bam
    samtools view -@ 50 -b -F 2 $outdata${i}.bam > unmapped/${i}_umapped.bam.
58. Split unmapped bam into fastq.
  a. For taxonomic classification mapped reads need to split as fastq file.
  b. Kraken2 takes paired fastq files as input.
    samtools fastq -@ 50 $outdata/unmapped/${i}_umapped.bam −1 $outdata/bam2fastq/${i}_R1.fastq.gz −2 $outdata/bam2fastq/${i}_R2.fastq.gz −0 /dev/null -s /dev/null -n.
  c. Output.

**Figure:**
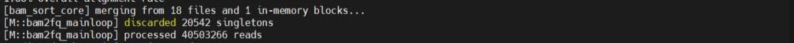
59. Taxonomic classification.
  a. Taxonomic classification with Kraken2.
    i. Installation of kraken2.
    ii. Download database here.
      https://lomanlab.github.io/mockcommunity/mc_databases.html.
    iii. Download the database from the Kraken2 website, which contains bacteria, archaea, and viral reference sequences.
    iv. Kraken2 map reads with taxa using k-mers from the genomic database and assigns taxonomy.
    v. Run:
      kraken2 --db $index --report $outdata/kraken2/k2_report/${i}.report --output $outdata/kraken2/${i}.output --gzip-compressed --paired $outdata/bam2fastq/${i}_R1.fastq.gz $outdata/bam2fastq/${i}_R2.fastq.gz.
    vi. Output:

**Figure:**
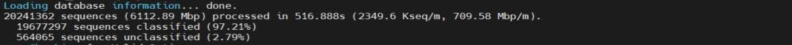

      ***Note:*** Although, kraken2 provides faster and more accurate classification, however, it cannot assign each read to the species. To overcome this limitation, we analyzed Kraken2 output with Bracken2. The provided screenshot illustrates the increase in reads identified by Bracken2 compared to those assigned by Kraken2 (reads assigned to a specific species in a particular sample).

**Figure:**
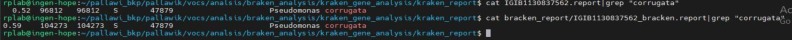
  b. Taxonomic classification with Bracken.
    i. Bracken2 (Bayesian Re-estimation of Abundance after classification with KrakEN) uses the taxonomy assigned by Kraken2 to estimate the number of reads per sample that come from different species, using the Kraken report output file.
    ii. Bracken2 provides faster and more accurate classification.
    iii. Run:
      bracken -d $index -i $outdata/kraken2/${i}.report -o $outdata/braken2/${i}.bracken.
    iv. Output:

**Figure:**
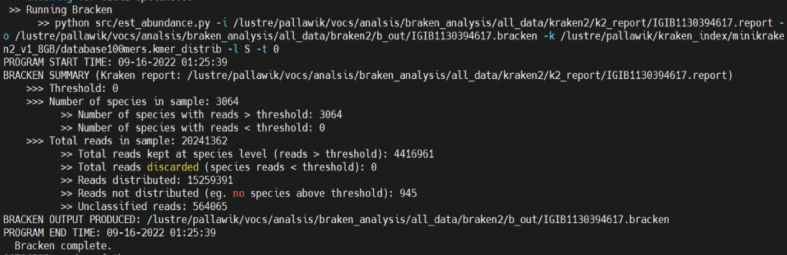
      Output for one complete loop

**Figure:**
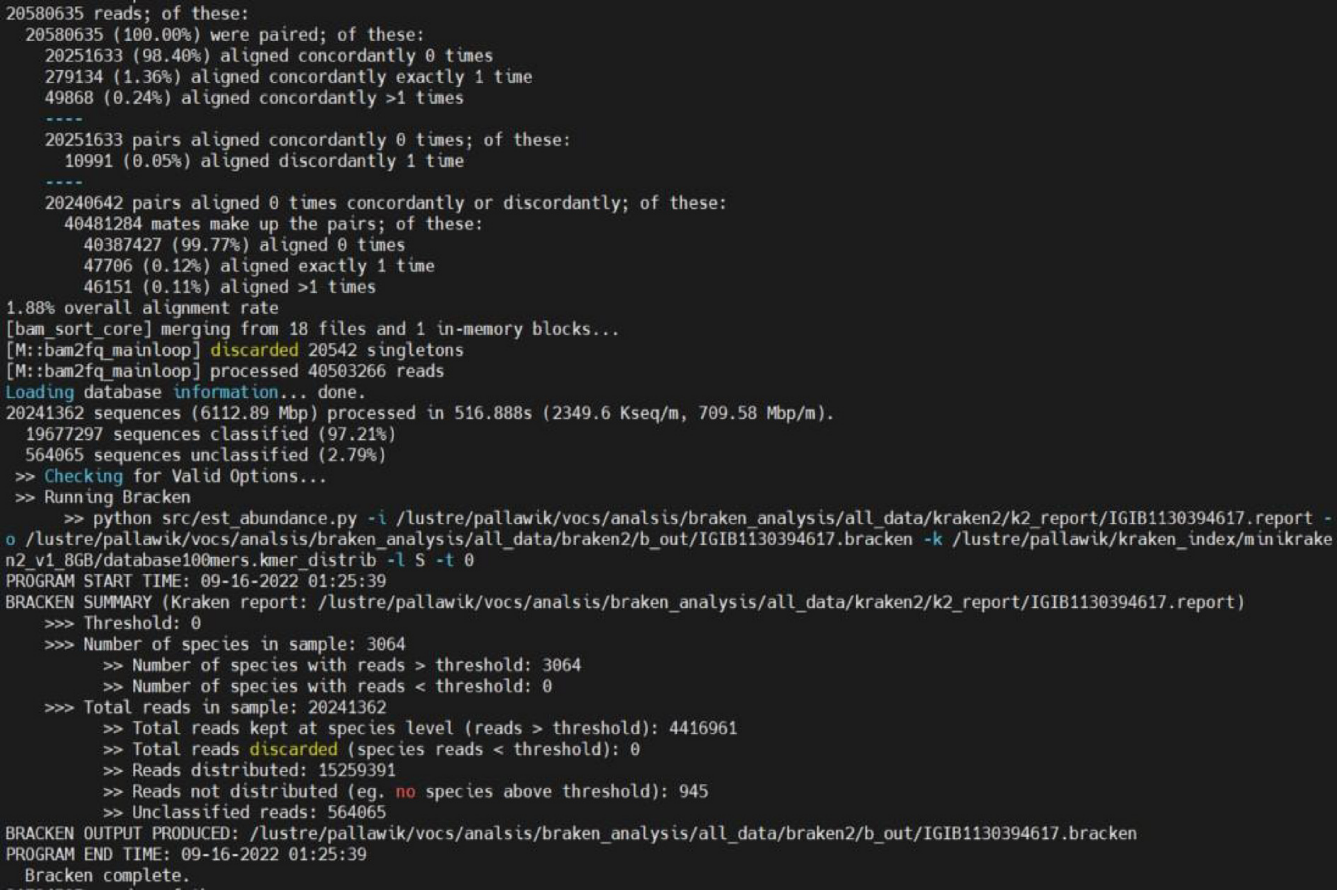
      Downstream Analysis
60. Normalisation.
  a. To normalize the sampling depth, use the CSS (Cumulative Sum Scaling: median-like quantile normalization) method from R-package metagenomeSeq.
  b. Downloading and installing metagenomeseq.
    if (!requireNamespace("BiocManager", quietly = TRUE))
    install.packages("BiocManager")
    BiocManager::install("metagenomeSeq")
    library(metagenomeSeq)
  c. Run:
    norm_voc <- read.csv(file = "voc/kraken/bracken-out.csv")
    norm_voc_1 <- norm[ ,c(2:ncol(norm_voc))]
    metaSeqObject = newMRexperiment(norm_voc_1)
    metaSeqObject_CSS = cumNorm( metaSeqObject , p = cumNormStatFast(metaSeqObject) )
    read_count_CSS = data.frame(MRcounts(metaSeqObject_CSS, norm = TRUE, log = TRUE))
    rownames(read_count_CSS) <- norm$species
    write.csv(read_count_CSS, "voc/normalized_bracken_voc.csv")
61. Alpha and Beta diversity.
  a. Execute alpha and beta diversity analyses in R (4.2.0) using vegan (v2.6–2) (Dixon, 2009) and Phyloseq (v1.40.0).
  b. For alpha diversity: Use the estimated richness function (Shannon’s, and Chao-1) from the phyloseq package.
  c. For beta diversity: Use the phyloseq::distance from the vegan package to generate Bray-Curtis dissimilarity matrix and principal coordinate (PCoA) values.
  d. After the analysis, export the data from R as a CSV file.
  e. For data plotting and visualization: Use the library Seaborn (0.12.2) and Matplotlib (3.1) build on Numpy (1.11.0) in Python (3.6). The detailed scripts and documentation can be found in the GitHub repository: https://github.com/pallawikumari/visualization-in-python/tree/V1.0.0.

if (!require("BiocManager", quietly = TRUE))

install.packages("BiocManager")

BiocManager::install("phyloseq")

install.packages("vegan")

#Alpha diversity

library("vegan")

data_biom <- import_biom("kraken_out.biom", parseFunction = parse_taxonomy_default)

data.alpha <- estimate_richness(data_biom)

#Beta diversity

braycurtis <- ordinate(physeq = data_biom, method = "PCoA", distance = "bray", weighted = TRUE)

braycurtis.export <- as.data.frame(braycurtis$vectors, row.names = NULL, optional = FALSE, cut.names = FALSE, col.names = names(braycurtis.$vectors), fix.empty.names = TRUE, stringsAsFactors = default.stringsAsFactors())

write.csv(braycurtis.export, file = "braycurtis-krakenout-voc.csv")

percent_explained <- 100 ∗ braycurtis$values$Eigenvalues / sum(braycurtis$values$Eigenvalues)

percent_explained[1:5]

62. HUMAnN3 analysis.
  a. Use HUMAnN3 (The Human Microbiome Project Unified Metabolic Analysis Network 3) analysis pipeline to illustrate the microbial population of the metabolic pathways based on the MetaCyc database.
    conda create -n METAPH
    conda activate METAPH
    conda install -c bioconda humann
  b. Download the database using:
    humann_databases --download uniref uniref. 90_diamond.
  c. Run:
    input = /lustre/pallawik/fastq/merge
    output = /lustre/pallawik/humann3/humann_out
    chocophyln = /lustre/pallawik/humann3/db/chocophlan
    diamond = /lustre/pallawik/humann3/db/uniref
    for i in $(ls ∗.fastq.gz |rev | cut -c 10- | rev | uniq)
    do
    humann --threads 1000 --input $input/${i}.fastq.gz --output $output/${i} --remove-temp-output --nucleotide-database $chocophyln --protein-database $diamond
    done.
  d. Output:

**Figure:**
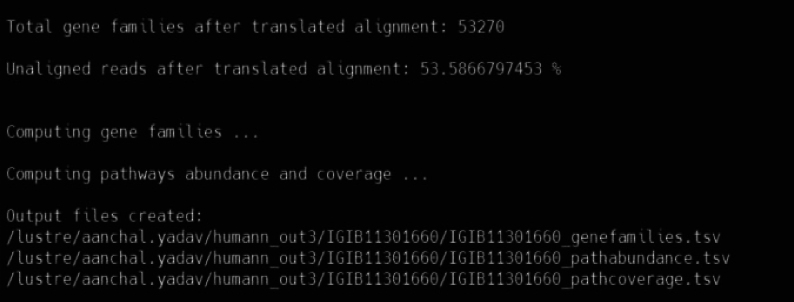
  e. Output Interpretation:
  f. Use Humann_renorm_table to obtain normalized pathway abundance from RPK (Reads per kilobase) to CPM (Counts per million) and join tables using humann_join_tables.
    humann_renorm_table --input ${i}_pathabundance.tsv --output output/${i}_pathabundance_relab.tsv --units relab
    humann_join_tables --input humann-output/ --output humann_pathabundance.tsv --file_name pathabundance_relab
    humann_join_tables --input humann-output/ --output humann_pathcoverage.tsv --file_name pathcoverage
    humann_join_tables --input humann-output/ --output humann_genefamilies.tsv --file_name genefamilies_relab
    humann_barplot -i path_with_bac_test_barplot.tsv -f ANAGLYCOLYSIS-PWY -m condition --output ANAGLYCOLYSIS-PWY.png --remove-zeros --sort sum.
    ***Note:*** The tool (humann_barplot) is used to assess the abundance of a specific pathway, such as “ANAGLYCOLYSIS-PWY” as mentioned in the provided code block. It's important to note that this tool can be employed to visualize abundance of various pathways, not limited to just one.

### Transcriptome analysis

**Timing: 1–2 days (according to the availability of computational resources, may take less time)**

63. Indexing Transcriptome and Quantifying Reads using Salmon.
  a. Download reference transcriptome in FASTA format for the Protein-coding transcript sequences on the human reference from (https://www.gencodegenes.org/human/).
    wget https://ftp.ebi.ac.uk/pub/databases/gencode/Gencode_human/release_44/gencode.v44.pc_transcripts.fa.gz
    gzip -d gencode.v44.pc_transcripts.fa.gz.
  b. Install the Salmon on the working directory of the server. That can install using the conda package manager or download it from the official Salmon website e.g., SALMON-v.2.2.0.tar.gz (https://salmon-tddft.jp/download.html) for RNA-seq read quantification.
    conda install bioconda::salmon.
  c. Indexing the Transcriptome: In terminal navigate to the directory containing the transcriptome FASTA file and execute the below command.
    salmon index -t gencode.v44.pc_transcripts.fa -i index.
  d. Quantify Reads using Salmon: Navigate to the directory where RNA-seq data is located. With the below command Salmon will produce several output files, including quantification results. Key files include "quant.sf" containing transcript-level abundance estimates.
    for i in ∗_R1.fastq.gz; do n = ${i%.∗}; echo "salmon quant -i /home/ranjeet.maurya/reference/index/ -l A −1 $i −2 ${i%%_∗}_R2.fastq.gz -p 300 --validateMappings -o quants/${i%%_∗}"; done > quant.sh
    bash quant.sh.
64. Differential expression analysis using DESeq2.
  a. Download the comprehensive gene annotation (GTF format) file (gencode.v40.annotation.gtf) for human reference GRCh38 from GENCODE (https://www.gencodegenes.org/human/).
  b. Install the following R packages for DESeq2 analysis.
    if (!require("BiocManager", quietly = TRUE))
    install.packages("BiocManager")
    BiocManager::install("tximport")
    install.packages("dplyr")
    BiocManager::install("GenomicFeatures")
    install.packages("readr")
    BiocManager::install("DESeq2″)
  c. Load the above installed R packages using the following R code.
    setwd("/path/to/quant_files")
    library(tximport)
    library(dplyr)
    library(GenomicFeatures)
    library(readr)
    library(DESeq2)
    library(RColorBrewer)
    library(ggplot2)
  d. Load Sample tables and quantification files.
    txdb <- makeTxDbFromGFF("gencode.v40.annotation.gtf")
    k <- keys(txdb, keytype = "GENEID")
    tx2gene <- select(txdb, keys = k, keytype = "GENEID", columns = "TXNAME")
    head(tx2gene)
    write.csv(as.data.frame(tx2gene),file = "gencode.v40.annotation.csv")
  e. Run DESeq2 with the settings comparing condition A vs. condition B.
    tx2gene<-read.csv("gencode.v40.annotation.csv", header = TRUE)
    samples <- read.csv("group1-vs-group2-samples.csv", header = TRUE)
    #Here, the creation of the “samples” object is importing a sample sheet where the ‘Group’ contains the variables denoting the ‘group1’ and ‘group2’ that would be used to perform the differential gene-expression.
    files <- file.path(samples$Patient.ID, "quant.sf")
    names(files) <- paste0(samples$Patient.ID)
    txi.salmon <- tximport(files, type = "salmon", tx2gene = tx2gene)
    dds <- DESeqDataSetFromTximport(txi.salmon, samples, ∼Patient.ID)
    keep <- rowSums(counts(dds)) >= 10.
    #creation of the “keep” object is to find genes which are expressed above a certain expression value threshold.
    head(dds)
    dds
    dds <- dds[keep,]
    cts<-counts(dds)
    dd<-as.matrix(cts +1)
    head(dd)
    write.csv(dd, "count_data.csv", row.names = TRUE)
    dds <- DESeqDataSetFromMatrix (countData = dd, colData = samples, design = ∼Group)
    dds<-DESeq(dds,)
    head(dds)
    res <- results(dds,contrast = c("Group", "group1", "group2")))
    head(res)
    summary(res)
  f. Write and save the results.
    res<-res[order(res$padj),]
    head(res)
    write.csv (res, "DEGs-group1-vs-group2.csv")
    ***Note:*** This will save the differential expression results to a comma-separated file (csv) file for further analysis or visualization. The analysis outcome will produce a CSV file that presents the outcomes of the differential expression tests for all genes, ordered by the adjusted p-value. The columns in the file will encompass the mean expression, fold change between conditions, relevant statistical test metrics, p-value and adjusted p-value. The adjusted p-values are derived from the p-values and should be used in preference over the p-values.

### Pathway enrichment analysis

**Timing: 1–2 h**

65. Functional enrichment analysis using clusterProfiler and KEGG pathways.
  a. Install and load “clusterProfiler” R package.
  b. Consider pathways with a *p value* cutoff of 0.05.

# Step 1: Install the 'ClusterProfiler' package

install.packages("ClusterProfiler")

# Step 2: Install the 'org.Hs.e.g.,.db' Bioconductor package

if (!requireNamespace("BiocManager", quietly = TRUE))

install.packages("BiocManager")

BiocManager::install("org.Hs.e.g.,.db")

# Step 3: Load required libraries

library(ClusterProfiler)

library(org.Hs.e.g.,.db)

# Step 4: Read data from CSV file and select relevant columns for Ensembl Ids and Fold change

df <- read.csv("DEGs-moderate-vs-mild.csv")[,c(1,3)]

ids <- bitr(df$Ensembl.Ids, fromType = "ENSEMBL", toType = "ENTREZID", OrgDb = "org.Hs.e.g.,.db")

dedup_ids = ids[!duplicated(ids[c("ENSEMBL")]),]

df2 = df[df$Ensembl.Ids %in% dedup_ids$ENSEMBL,]

# Step 5: Map Entrez IDs to the dataframe

df2$Y = dedup_ids$ENTREZID

kegg_gene_list <- df2$log2FoldChange

names(kegg_gene_list) <- df2$Y.

# Step 6: Create a gene list with log2FoldChange values

kegg_gene_list<-na.omit(kegg_gene_list)

kegg_gene_list = sort(kegg_gene_list, decreasing = TRUE)

# Step 7:Perform KEGG pathway enrichment analysis

gse <- gseKEGG(geneList = kegg_gene_list,

organism = "hsa",

nPerm = 10000,

minGSSize = 3,

maxGSSize = 800,

pvalueCutoff = 0.05,

pAdjustMethod = "BH",

keyType = "ncbi-geneid")

# Step 8: Write the results to a CSV file

write.csv(gse@result, "group1-vs-group2_kegg_enrich.csv")

### Correlation analysis

**Timing: 30 min**

Use this analysis to identify the correlation between bacterial species and the expressed host genes.

66. Utilize microbial species for correlation analysis with differentially expressed host genes using the corrr (0.4.4) package in R, employing Spearman’s correlation analysis.
67. Establish a correlation coefficient cut-off of ≥±0.8 and a p-value of ≤0.05 to construct a correlation plot and visualize the interaction network in Cytoscape (version 3.9.1), a GUI-based tool.

library(dplyr)

library(tibble)

library(tidyr)

library(magrittr)

library(corrplot)

library(RColorBrewer)

library(corrr)

my_data <- read.csv("gene.csv")

bacteria <- read.csv("bacteria.csv")

df <- bind_cols(bacteria[, −1],my_data[, −1])

res1 <- cor.mtest(df, conf.level = 0.95)

res2 <- res1$p %>% as.data.frame()

rownames(res2) <- colnames(df)

colnames(res2) <- colnames(df)

res2 <- res2%>%

rownames_to_column("rowname") %>%

gather("var", "p", -rowname)

A < - corrr::correlate(bind_cols(my_data[, −1], bacteria[, −1])) %>%

pivot_longer(cols = -term,names_to = "var",values_to = 'value') %>%

rename(rowname = term) %>%

left_join(res2) %>%

mutate(value = ifelse(*p* < 0.05, NA_integer_, value)) %>%

select(-p) %>%

spread(var, value) %>%

filter(rowname %in% colnames(my_data)) %>%

select(one_of(c("rowname", colnames(bacteria)[-1]))) %>%

as.data.frame() %>%

column_to_rownames("rowname") %>%

as.matrix() %>%

corrplot::corrplot(is.corr = FALSE, na.label.col = "white",

col = brewer.pal (*n* = 10, name = "PuOr"), tl.col = "black", tl.srt = 45)

## Expected outcomes

The major outcome of this protocol involves the identification of Transcriptionally Active Microbes (TAMs) and their integration with host-response genes. Through an integrative approach of dual RNA-Sequencing and metagenomic analysis, this method provides new insights into potentially functional host-microbiome dynamics and explores the association between host pathways, genes, and functional microbial species. This method offers an effective means of identifying putative microbial candidates’ vis-à-vis various diseases caused by a primary infecting pathogen.

## Limitations

The RNA-seq protocol has been fine-tuned to work best with a substantial input of total RNA, typically ranging from 0.1 to 1 μg. This range offers an ideal opportunity to include a diverse range of RNA samples. However, it’s crucial to optimize the protocol initially to ensure that each sample is consistently processed with the same RNA concentration. This approach eliminates any potential bias in further steps of library preparation. This is even more important as we undertake integrative analysis for the expressed host genes and the transcribed microbes in those cohorts of patients.

Moreover, one of the protocol’s key steps involves removing rRNA to enrich the desired mRNA reads. This step significantly increases the coverage of the desired mRNA transcripts. Unfortunately, it’s observed that not every sample in our library preparation process achieves uniform rRNA depletion. This discrepancy results in some more reads being assigned to rRNA in few samples. This protocol focuses mainly on the coding mRNA and long non coding RNAs leaving the small mRNAs.

Additionally, during the purification step of adapter ligation, if not executed meticulously, there’s a risk of adapter dimer formation. These adapter dimers can be problematic for the success of the library preparation process. If not adequately removed, they may lead to issues in our final library.

## Troubleshooting

### Problem 1

Insufficient rRNA depletion (related to Step 45).

### Potential solution

• Make sure that the rRNA removal beads (RRB) are at room temperature before adding it to the sample and do not allow the RRB pellets to dry.
• Pipette up and down quickly to ensure thorough mixing. Insufficient mixing leads to lower levels of rRNA depletion.
• Pipette with the tips at the bottom of the well to prevent foaming. Excess foam leads to sample loss because foam does not transfer efficiently.

### Problem 2

Improper RNA fragmentation (related to Step 45t).

### Potential solution

• The fragmentation of nucleic acids is a crucial step in optimizing library preparation, clustering, and sequencing, and the specific conditions for fragmentation depend on the initial state of the RNA, whether it’s intact or degraded.
• When working with intact RNA, the TruSeq Stranded Total RNA Library Prep protocol for transcriptome analysis involves fragmenting the RNA after rRNA depletion. For samples containing degraded RNA, it’s important to avoid over-fragmentation. In such cases, the fragmentation time should be reduced.
• This can be achieved by either omitting or modifying the thermal cycler Elution 2-Frag-Prime program to involve a 94°C incubation for X min, followed by a 4°C hold.

### Problem 3

Adapter Dimers formation (related to Step 51b).

### Potential solution

• A Bioanalyzer chip is run to check the adapter dimers in the library which hampers the library binding to the flow cell. So to remove dimers before putting sequencing is inevitable and for this 1:1 AMPure beads purification is done for libraries which have adapter dimers in BA profile.

### Problem 4

Storage issue during HUMAnN3 analysis (related to Step 62).

### Potential solution

In HUMAnN3, intermediate files are generated during the analysis which need a vast amount of space (30–40 GB/sample). Removing those intermediate files will be helpful if there is limited storage availability.

### Problem 5

Potential error during DESeq2 analysis (related to Step 64).

• Error: 'counts' must be a numeric matrix.
• Error: all genes have equal values across samples.
• Error: sample names in colData must be the same as colnames in countData.

### Potential solution

Performing DESeq2 analysis can sometimes lead to errors due to various reasons such as issues with data format, missing values, or incorrect input.

• This error occurs if the input data for DESeq2 is not a numeric matrix. Make sure your count data is in a numeric matrix or data frame. For example, if your counts are in a table named countData, you can convert it to a matrix using the “as.matrix” function.
• This error may occur if all counts for a particular gene are the same across all samples. Check your data for such cases and consider filtering out genes with very low variance.
• Ensure that the sample names in your colData match the column names in your count data. It’s crucial that they are in the same order.

### Problem 6

The protocol demonstrated here uses a large compute resource, which might not be and often not feasible for many researchers/ groups.

### Potential solution

• All this analysis can also be done with a computer with ∼16–32 GB of RAM, only the execution time will increase. Instead of servers, laptops or personal systems can also be used for analyses utilizing alternate installation methods.
• Apart from this, alternatively the user can use web-based analysis platforms, such as Galaxy server (https://usegalaxy.org/), where tools are available and easy to execute.

## Resource availability

### Lead contact

Further information and requests for resources and reagents should be directed to and will be fulfilled by the lead contact, Rajesh Pandey (rajeshp@igib.in; rajesh.p@igib.res.in).

### Technical contact

Technical questions on executing this protocol should be directed to and will be answered by the technical contact, Rajesh Pandey (rajeshp@igib.in; rajesh.p@igib.res.in).

### Materials availability

This study did not generate new unique reagents and material.

### Data and code availability

• RNA-seq data have been deposited at NCBI SRA, and are publicly available as of the date of publication. The accession numbers for the RNA-seq data reported in this paper are NCBI SRA: PRJNA676016, PRJNA678831 (Pre-VOC), PRJN868733, PRJNA952815 (VOCs). Accession numbers are listed in the key resources table.
• The software code and detailed script used by this protocol (for data plotting and visualization) can be accessed at https://github.com/pallawikumari/visualization-in-python/tree/V1.0.0 and has been deposited at Zenodo (https://zenodo.org/doi/10.5281/zenodo.10983961).
• This paper does not report original code.
• Any additional information required to reanalyze the data reported in this paper is available from the lead contact upon request.
